# Supplementary material for: Effects of COVID-19 disease on PAI-1 antigen and haematological parameters during disease management: A prospective cross-sectional study in a regional Hospital in Ghana
Source: PLOS Glob Public Health. 2023 Jun 22;3(6):e0001866. doi: 10.1371/journal.pgph.0001866 (PMC10286987; doi:10.1371/journal.pgph.0001866)
Supplement: Attachment — Submitted filename: RESPONSE TO REVIEWERS.docx [file pgph.0001866.s001.docx]

Dear Editor,

Thank you for your email. I am pleased to resubmit manuscript titled “***Effects of COVID-19 disease on PAI-1 antigen, haematological parameters and vital signs during disease management: A prospective cross-sectional study in a regional Hospital in Ghana***“for your consideration.

Authors do appreciate your endless efforts to ensure improvement in the manuscript.

The concerns raised by the editorial team have been addressed and highlighted below and marked in the respective sections in the manuscript. I look forward to your favourable response.

Thank you.

**The manuscript has been edited for language usage, spelling and grammar by Grammarly Inc. USA.**

**General Comments**

1. We have noticed that you have uploaded Supporting Information files, but you have not included a list of legends. Please add a full list of legends for your Supporting Information files after the references list.

Response: List of legends for the Supporting Information files have been included, after the References list

**Reviewer #1:**

**General comments:**

1. Sentences are too long and some of them are mixed and therefore authors should ensure that they make short sentences without losing the meaning (see sentences 66-73)

Sentences have been revised for clarity

2. Some sentences are not written in full and so authors need to check the grammar and the sentence construction especially in the results section

Sentences have been revised accordingly

3. Authors are too many and what was the contribution of each of the authors in this work

The contributions of all authors have been inserted accordingly.

This work was carried out in collaboration between all authors. Authors CN, MO, LDA, YQ, CPE, A-WI, AM and EBA conceived and designed the study. Authors CN, KM, SKA, YQ, FO-B, GA, SD, CAD, FAA, VA, MO, SBB, and EBA wrote the protocol and the first draft of the manuscript. Authors LDA, EBA, CPE, YQ, CAEW, SA, A-WI, and AM participated in the data collection. Authors LDA, DAA, CN, EBA, CPE, A-WI, SM, BT, AM, WAG, PEA, PA, CAEW, RAYA and SA performed the laboratory works. Authors CN, KM, SKA, MO, YQ, EMA, and FO-B analysed and interpreted the data. Authors CN, LDA, SBB, YQ, CPE, A-WI, AM EMA, PEA, KM, DAA, SA and SKA managed the literature searches. Authors CN, MO, SKA, GA, SD, KM, LDA, FO-B, YQ, CAD, FAA, VA, SBB, EBA, CPE, SM, BT, EMA, WAG, PA, CAEW, and SBB drafted the manuscript. All authors critically reviewed, revised and approved the final manuscript.

**Specific comments:**

4. Title does not reflect the work done and so I suggest to change it to "Effects of COVID-19 disease on PAF-1 antigen, hematological parameters and vital signs during disease management: A prospective cross-sectional study in a regional Hospital in Ghana"

Authors appreciate the suggestion. The title of the study has been revised to reflect the work done as recommended.

5. Line 57: insert antigen after word PAI-1

“Antigen” has been inserted after the word “PAI-1”

6. Line 66-74: Results should be presented as means (CI at 95% and then the p-values. Otherwise just presenting the p-values does not reflect good presentation of the results. Secondly at what confidence intervals were these p-values computed.

This has been revised accordingly to reflect either the mean or median depending on the nature of the data. The data were computed at 95% CI and this has been captured in the “Materials and methods” session.

7. Line 76: Use past tense and mot present tense and so change “is” to “was”

The word “is” has been replaced with “was”

8. Line 100: It is too long and it addresses the global burden of COVID-19 and then in Ghana. The sentence should be split up.

The sentence has been split into two

9. Line 103: Insert “is” after COVID-19

The word “is” has been inserted after COVID-19

10. Line 104: Insert “and this virus” after (SARS-CoV2)

“and this virus” has been inserted after (SARS-CoV-2)

11. Line 108: Replace word “suggested” to “reported” and authors need to explain more on the “Cytokine storm”

The word “reported” has replaced the word “suggested”. Also cytokine storm has been described as the “rapid release of excessive cytokines” in the next line

12. Line 113: What are the main cytokines involved in the cytokine storm?

TNF-α, IL-1, and IL-6 are mostly involved

13. Line 117-121: Sentence is too long and need to be split up since the meaning is lost

This has been revised for clarity

14. Line 127: Sentence need to be paraphrased

This has been revised accordingly

15. Line 136: Which enzyme has not been studied? And suggest the authors replace “our” with “present”

This has been addressed. PAI-1 enzyme has been clarified, and the word “our” has been replaced with “present”

16. Line 138: insert “of the COVID-19 disease in the infected individuals” after word “complications”

This has been addressed to reflect the suggestions made

17. Line 140: Should be “Materials and methods” other than “Methodology”

The heading has been changed from “Methodology” to “Materials and methods”

18. Line 141: Should be “Study design and setting”

This has been revised accordingly

19. Line 142: Insert “that was conducted” after word “study”

This has been addressed as suggested

20. Line 147: Replace “surrounded “with “surrounding”

The word “surrounding” has replaced the word “surrounded”

21. Line 149: Can authors provide a map of the study site if possible where the study was conducted!!

The map of the study site has been inserted as recommended

22. Line 154: Authors should include a statement of the “Helsinki declaration on use of human subjects in research”

The Helsinki declaration on research involving humans has been inserted

23. Line 152: Replace word “this” with “the”

The word “this” has been replaced by the word “the”

24. Line 155: It should read “Study population”

The heading has been revised to read ‘Study population”

25. Line 173: Authors insert a sub-section “Study participant selection criteria” and then also separate the “Inclusion and Exclusion criteria” What was the age ranges of the study participants that were supposed to be included in the study? What happened to those who refused to consent or those who were severely ill and could not sign the consent form? What about those who were below 18 years of age? What happed to those who died and were not able to get the second set of data or there was no death recorded among those who were recruited?

The sub-heading “Study participants’ selection criteria” has been inserted.

“Inclusion and exclusion criteria” has been separated

The exclusion criteria has been revised to contain those who refused to give their consents.

There was no restriction on age limits, however, during the study period, only adults (21 to 81 years) reported to the hospital with COVID-19. Again, none of the COVID-19 patients was critically ill so all could give their consents. Also, none of the participants died during the study period. Hence, the second set of data were successfully collected from all the participants recruited.

26. Line 177: Include “Data collection and sources of data”

This has been addressed accordingly

27. Line 182: What are those commercial tubes and their names!!!

This has been revised. The swabs were collected into sterile tubes containing virus transport medium

28. Line 183 -186: Sentence is too long and please split it up.

The sentence has been split into two

29. Line 187: What was the rationale of using 2 anticoagulants i.e. K2EDTA and sodium citrate??

K_2_EDTA is often the most appropriate anticoagulant for FBC measurement. Also, sodium citrate is mostly preferred for coagulation analyses, and the manufacture recommended the citrate as first choice anticoagulant for the PAI-1 assay.

30. Line 195: What do authors mean “on daily basis” when they had to samples to be collected? Please clarify.

Authors meant that blood samples for FBC were not stored, but assayed on same day of sample collection. So on daily basis, ones participants blood were collected, FBC were measured without storing the blood.

31. Line 203 -204: WHO classifies the COVID-19 disease progression? Please use that classification

Authors adopted the WHO’s guidelines on the severity of COVID-19 as contained in their

“Clinical management of COVID-19: Living guideline, 13 January 2023” document [26].

32. Line 212: Insert “FBC) after “counts”

“FBC” has been inserted after the word “counts”

33. Line 228: Which “Lysing agent was used”?

The lysing agent used was “cyanide-free sodium lauryl sulphate (SLS) reagent”. This has been inserted into the main text

34. Line 243-251: Many tests were used. Of these tests, which test was performed on which data?

This has been addressed accordingly as both parametric and non-parametric data have been specified in the data analysis section.

Parametric data (pulse, SPO2%, blood pressure, HCT%, MCV, granulocytes and MPV) were presented in means ± standard deviation and non-parametric data (age, temperature, Hb, RBC, MCH, MCHC, RDW-CV, WBC, lymphocyte count, platelet, PDW%, PCT%, ferritin and PAI-1) in medians (1st-3rd quarters). Unpaired parametric data were appropriately compared with Student’s T-test orand unpaired non-parametric data compared with Mann-Whitney U-test. Paired parametric data (on admission and after recovery) were compared using the Paired Sample T-test, whilst paired non-parametric data were compared with Wilcoxon signed-rank test.

35. Line 255: the measure is percentage (%), I propose that authors when presenting their results be presented as “% (n) all through. Even some of the results presented are confusing line on line 255. I also suggest of normal reference values are included to see the variation form the normal during the disease progression and recovery. I also suggest that “units” be put in brackets for each parameter

This has been addressed accordingly. Presentation of the results has been revised, normal reference values added as legends to each table and figure, and all units have been put in brackets for each parameter.

36. Line 262-265: Please construct full sentences.

This has been revised

37. Tables of the results: Are these results presented as “means, CI, P-values? Or as single value measured for a single sample? See all the tables. Also see comment on Line 255

Parametric data (pulse, SPO_2_%, blood pressure, HCT%, MCV, granulocytes and MPV) were presented in means ± standard deviation, whilst non-parametric data (age, temperature, Hb, RBC, MCH, MCHC, RDW-CV, WBC, lymphocyte count, platelet, PDW%, PCT%, ferritin and PAI-1) were presented in medians (1^st^-3^rd^ quarters). Again, categorical data were presented in frequencies with their corresponding percentages

38. Line 279 – 294: Construct sentences for each parameter and not mixing them

The FBC parameters have been clearly compared between severe and non-severe COVID-19 participants.

39. Line 311: Figure 1 and figure 2 cannot be seen and if so those figures and not clearly labeled to be distinguished.

As required, the figures have been uploaded as separate files at PACE (<https://pacev2.apexcovantage.com/Upload>)

40. Discussion:
41. Sentences are too long and they need to be split up.

The discussion section has been revised accordingly

42. Line369: Sentence is not clear and it need to ne paraphrased

This sentence has been paraphrased for clarity

43. Conclusion:
44. Line 470: use past tense and not present tense

This has been revised as recommended

45. Line 479: Grammar typo and it should be corrected i.e. “appreciater”

This word has been replaced with the word “appreciate”

**Reviewer #2:**

The results of this important and robust cross sectional study contribute towards the growing body of evidence required for development of new therapeutic interventions for management of the COVID-19 pandemic. Research and development of new drugs to treat COVID-19 and related comorbid conditions is still work in progress, results from this study inform the current management policies and guidelines, and is critical for future large scale clinical research studies.

Response: Authors are grateful and appreciate the comments
