# Supplementary material for: Effects of COVID-19 disease on PAI-1 antigen and haematological parameters during disease management: A prospective cross-sectional study in a regional Hospital in Ghana
Source: PLOS Glob Public Health. 2023 Jun 22;3(6):e0001866. doi: 10.1371/journal.pgph.0001866 (PMC10286987; doi:10.1371/journal.pgph.0001866)
Supplement: Attachment — Submitted filename: RESPONSE TO REVIEWERS.docx [file pgph.0001866.s002.docx]

Dear Editor,

Thank you for your email. I am pleased to resubmit manuscript titled “**Serum anti-erythropoietin antibodies among pregnant women with *Plasmodium falciparum* malaria and anaemia: A case-control study in northern Ghana** “for your consideration.

Authors do appreciate your endless efforts to ensure improvement in the manuscript.

The concerns raised by the editorial team have been addressed and highlighted below and marked in the respective sections in the manuscript. I look forward to your favourable response.

Thank you.

**The manuscript was edited for language usage, spelling and grammar by Grammarly Inc. USA.**

**General Comment:**

1. Figure: please (a) provide a direct link to the base layer of the map (i.e., the country or region border shape) and ensure this is also included in the figure legend; and (b) provide a link to the terms of use / license information for the base layer image or shapefile. We cannot publish proprietary or copyrighted maps (e.g. Google Maps, Mapquest) and the terms of use for your map base layer must be compatible with our CC-BY 4.0 license.

Response: The map showing the study site has been revised to suit the requirements.

**Reviewer #1:**

1. Line 200: K2EDTA tubes for full blood counts (FBC) – tubes should be purple top vacutainers!!!!!

Response: This has been revised to contain the “purple top vacutainers” as suggested

2. Line 204- 205: Blood samples in the plain tube (after sufficient clotting) and citrate tube were spun to separate the respective serum and plasma from the cells --- Centrifuged and Model of centrifuge and at what gram-force?

Response: The word **“spun”** has been replaced with **“centrifuged”.** The centrifuge’s model and country of origin as well as the speed of centrifugation have been inserted accordingly **[centrifuged (CENTRIFUGE 80–1, Japan) at 3000 rpm for 15 minutes]**

3. Table 1: For age and gender, the findings should be reported as % (N) and not vice versa as it is reported in the text.

Response: This has been addressed accordingly.
