# Supplementary material for: Effects of COVID-19 disease on PAI-1 antigen and haematological parameters during disease management: A prospective cross-sectional study in a regional Hospital in Ghana
Source: PLOS Glob Public Health. 2023 Jun 22;3(6):e0001866. doi: 10.1371/journal.pgph.0001866 (PMC10286987; doi:10.1371/journal.pgph.0001866)
Supplement: Attachment — Submitted filename: AUTHORS RESPONSE TO REVIEWERS.docx [file pgph.0001866.s003.docx]

Dear Editor,

Thank you for your email. I am pleased to resubmit manuscript titled **“Effects of COVID-19 disease on PAI-1 antigen and haematological parameters during disease management: A prospective cross-sectional study in a regional Hospital in Ghana”** for your consideration.

Authors do appreciate your endless efforts to ensure improvement in the manuscript.

The concerns raised by the editorial team have been addressed and highlighted below and marked in the respective sections in the manuscript. I look forward to your favourable response.

Thank you.

**The manuscript was edited for language usage, spelling and grammar by Grammarly Inc. USA.**

**Reviewer #1:**

1. Table 1: Information in column of Non-severe for female shifted and added itself to the male. Let it be rectified. Otherwise am okay with it now.

Response: Authors are grateful for the observation. The information in the column of Non-severe for female has been rightly placed now

**Reviewer #3:**

I have gone through the revised manuscript by Nkansah et al.., unfortunately none of the issues I had raised has been addressed. The current uploaded letter to the editor by the authors is titled “I am pleased to resubmit manuscript titled “Serum anti-erythropoietin antibodies among pregnant women with Plasmodium falciparum malaria and anaemia: A case-control study in northern Ghana “for your consideration”. “Whereas the current manuscript is talking about Effects of COVID-19 disease on PAI-1 antigen, haematological 1 parameters and vital signs during disease management: A prospective cross-sectional study in a regional Hospital in Ghana”.

Response: Authors duly accept the mistake in the title of the manuscript in the letter to the editor and apologize for that. The title has now been revised to suit the current study as **“Effects of COVID-19 disease on PAI-1 antigen, haematological parameters and vital signs during disease management: A prospective cross-sectional study in a regional Hospital in Ghana”**

Other comments have been addressed below

**Title:**

1. The title is not very clear especially when they state “and vital signs during disease management:

What are these vital signs during disease management?

Response: The title of the manuscript has been revised, with the “vital signs” deleted

**Abstract**

2. PAI-1 Spell out the full term at its first mention, indicate its abbreviation in parenthesis and use the abbreviation from then on.

Response: This has been addressed accordingly

3. The aim should be properly formulated since the study was looking at the impact of COVID-19 disease on PAI-1 antigen, haematological parameters and vital signs during disease management:

Response: The aim has revised to include haematological parameters

4. Statistical analysis statement should be deleted from the abstract, this section is taken care of at the methodology section.

Response: Only the software for the data analysis (SPSS) and the significance level were mentioned in the methodology section of the abstract

5. The results should be summarized to *a*ddress the meaning of the findings and the importance of the current study. The way they are currently presented is confusing…

Response: The results section has been revised

6. The conclusion the authors states that Successful recovery from COVID-19, with reduced inflammatory response restores the haematological parameters. The authors are making this expansive conclusion, yet they are not attributing the inflammation to ferritin. This statement need to be clarified.

The conclusion section has been revised to include “as observed in the decline of plasma ferritin levels”

**Methodology section**

7. In line 249-255 the authors should state the absorbance wavelength at which the Plasma plasminogen activator inhibitor-1 and serum ferritin were measured.

Response: Both plasma PAI-1 and serum ferritin were measured at a wavelength of 450 nm. This has been inserted in the methodology section accordingly

**Results section**

8. In line 280…. The authors should delete one full stop

Response: Authors appreciate the mistake, and deleted one of the full stops

9. In Table one the variables Age (year and categories), and gender were determined by descriptive statistics in terms of percentages, however the authors goes ahead to indicate the P values, in my opinion this is misleading because no inferential statistical was applied for this variables..

Response: The information for the age (years and categories) and gender, even though were descriptives, were compared between the severe and non-severe COVID-19 participants. Statistically, the 2x2 table between gender and the COVID-19 severity is compared using Fisher’s Exact Test. The more than 2 categories (age in years category) was compared rightly between the COVID-19 severity groups by ANOVA

**Discussion**

10. **In line 416-418 The authors indicate that “**increased exposure of females to the highly contagious SARS-CoV- 2 as the transmission is often by air droplet, and females relatively interact more compared to males”. Is this statement justifiable?

Response: This influence was suggested because the females within the study area (Sunyani) were usually crowded in the business areas of the city. This, the authors anticipated, enhanced transmission of the SARS-CoV-2 among females in the area compared to their male counterparts

11. The sentence in line 512-514 should be rewritten.

This sentence has been revised accordingly

**Conclusion**

12. In line 523-524 The authors by concluding that “Successful recovery from COVID-19, with reduced inflammatory response, restored the haematological parameters” without referring to ferritin is misleading.

The conclusion section has been revised to include “as observed in the decline of plasma ferritin levels”
