# Supplementary material for: Effects of COVID-19 disease on PAI-1 antigen and haematological parameters during disease management: A prospective cross-sectional study in a regional Hospital in Ghana
Source: PLOS Glob Public Health. 2023 Jun 22;3(6):e0001866. doi: 10.1371/journal.pgph.0001866 (PMC10286987; doi:10.1371/journal.pgph.0001866)
Supplement: Attachment — Submitted filename: AUTHORS RESPONSE TO REVIEWERS.docx [file pgph.0001866.s004.docx]

Dear Editor,

Thank you for your email. I am pleased to resubmit manuscript titled **“Effects of COVID-19 disease on PAI-1 antigen and haematological parameters during disease management: A prospective cross-sectional study in a regional Hospital in Ghana”** for your consideration.

Authors do appreciate your endless efforts to ensure improvement in the manuscript.

The concerns raised by the editorial team have been addressed and highlighted below and marked in the respective sections in the manuscript. I look forward to your favourable response.

Thank you.

**The manuscript was edited for language usage, spelling and grammar by Grammarly Inc. USA.**

**Reviewer #3:**

The statement statistical software for the data analysis (SPSS) and the significance level should be completely deleted from the abstract

Response: This statement has been deleted from the abstract as suggested

**Specific comments:**

1. Line 60: This cross-sectional study “**was”** conducted…..

Response: The word **“was”** has been inserted

2. Line 61: You can say “**patients”** instead of participants

Response: “Participants” has been replaced with **“Patients”**

3. Line 68: In the result section under abstract, you did not mention about the total number of Severe COVID-19 and non-severe COVID-19 out of 51 cases. Please include the numbers.

Response: Of the 51 participants recruited into the study, 78.4% (40) had non-severe COVID-19 whiles 21.6% (11) experienced a severe form of the disease. This has been included in the abstract.

4. Did you find any significant difference about the restoration of the haematological parameters in severe COVID-19 and non-severe COVID-19 group?

Response: This analysis has been done and inserted into the manuscript as Table 4

5. What is your recommendation regarding PAI-1 assessment? How PAI-1 assessment help physicians to treat patients in better way.

Response: This will enhance the early detection of probable thrombotic events and prompts Physicians to provide interventions to prevent thrombotic complications associated with COVID-19. This has been inserted in the manuscript accordingly

6. Line 130. Use reference at the end of this sentence.

Response: Reference [14] has been moved to the end of the statement

7. Line 139: based on “present investigations”….any meaning

Authors actually meant “based on our knowledge”. This has been clarified in the manuscript

8. Line 142: You have mentioned that you only enrolled severe COVID-19 patients. However, you compared results between severe and non-severe COVID-19 patients. Please clarify.

Response: The non-severe group has been inserted for clarity

9. Line 164: What types of Informed consent; written or verbal. Mention please.

Response: Participants either signed or thumb-printed the consent form before their inclusion into the study

10. Line 165: Please also add information about severe and non-severe COVID-19 patients.

Response: This has been considered

11. Line 182: Why only recruited 51 cases? Why not 50 or 100?

Response: Please, 51 COVID-19 cases were confirmed at the hospital during the study period, and authors included all in the study after they gave their consent.

12. Line 184: Why you are telling it “Convenient sampling technique”? Actually, you selected cases based on case definition.

Response: Yes, authors appreciate that participants were selected based on case definition. This has been addressed in the manuscript

13. Line 199: Immediately a COVID-19 patient was diagnosed “**by rRT-PCR”**

Response: “by RT-PCR” has been inserted appropriately

14. Line 210: A definition is needed to define “recovery status of covid-19”. No clinical sign or no rRT-PCR positive?

The statement has been revised to include **“after showing no clinical sign and no RT-PCR positive result”**

15. Line 274: Definition is required for severe and non-severe covid.

Response: Please the definition for severe and non-severe COVID-19 has been addressed in the Methodology (Detection of SARS-CoV-2) section as recommended by W.H.O.

**“Participants were diagnosed with non-severe COVID-19 when the nasopharyngeal swab contained SARS-CoV-2, but without any sign of severe or critical disease. Severe COVID-19 patients had SARS-CoV-2 and either oxygen saturation (SpO2) less than 90% on room air, signs of pneumonia or signs of severe respiratory distress. Patients tend to experience critical COVID-19 when in addition to the presence of SARS-CoV-2, there is either an urgent need for life-sustaining treatment, acute respiratory distress syndrome (ARDS), sepsis or septic shock [26]”.**

16. Line 278: 45.1% (23) ???

Response: This represents the number of participants with 30-39 years as indicated in the manuscript

17. Line 282: SpO2. Not properly written.

Response: Please the percentage (%) unit has been put in parenthesis

18. Table 2: Column 2. Not understandable to me. Is it mean and range?

Response: No please, RBC in column 2 is presented in Median (25^th^-75^th^ percentiles)

19. Line 343-356: You repeatedly showed results in both table and narrative section. In narrative, it should be summarized results of table.

Response: The results section has been revised for the narrative to contain summarized results of the table.

20. Line 387-390: In discussion section, you may compare your results (PAI-1 Ag levels) with other studies by showing statistics.

Response: This has been considered, and PAI-1 statistics have been inserted for clarity

21. Line 483-485: Please give some statistics instead of plain statement.
22. Response: This has been considered, and PAI-1 statistics have been inserted for clarity
23. I believe that this study had some limitations. It is useful for future studies to inform limitations.

Response: Limitations to the study has been addressed at the end of the discussion

24. “the hypofibrinolytic state plays a role in the development of thrombotic events” – this statement not from your study. So, you can remove this.

Response: Authors acknowledge the concern and has been considered. The statement has been deleted accordingly

25. “Plasma levels of PAI-1 should be assessed during the management of severe COVID-19 in Ghana” – why it is important and what is the application of this results?

Assessment of PAI-1 will enhance the early detection of probable thrombotic events and prompts Physicians to provide interventions to prevent thrombotic complications associated with COVID-19.
